# Expectations and Perceptions of Dutch Pharmacy Staff Regarding a New Framework for Continence Care: A Focus Group Study

**DOI:** 10.1177/11786329211033263

**Published:** 2021-07-23

**Authors:** Miranda C Schreuder, Henk van der Worp, Esther I Metting, Marco H Blanker

**Affiliations:** Department of General Practice & Elderly Care Medicine, University of Groningen, University Medical Center Groningen, The Netherlands

**Keywords:** Absorbent pads, focus groups, incontinence pads, pharmacy, qualitative research, urinary incontinence

## Abstract

Based on complaints that patients with urinary incontinence were not receiving the correct medical aids, the Dutch Ministry of Health, Wellbeing, and Sports requested further exploration. This resulted in a new framework based on considering individual activities of daily living when providing continence products. We aimed to explore the expectations of pharmacy staff regarding this new framework for continence care in the Netherlands and to establish the facilitators and barriers associated with that care. In total, 15 participants from 7 different pharmacies participated in 2 focus groups. Data analysis was by thematic content analysis. Pharmacy employees were positive about the idea of considering individual daily activities when providing continence products in the new framework, but they did have some reservations about the feasibility of implementation in daily practice. Barriers to optimal continence care included low reimbursement for patients with incontinence, especially with non-standard needs, and poor communication between the various stakeholders in continence care. Efforts must be extended to review the current reimbursement system and to change the policies and information provided by stakeholders in continence care, before the new framework will make a real impact in clinical practice.

## Background

The Dutch health care system has provisions to reimburse the costs of long-term continence products when prescribed for urinary incontinence by a physician. Depending on the policy of their insurance company, patients are expected to go to either a local pharmacy or a national postal order supplier to buy reimbursable products.^1,2^ In practice, there is also cooperation between postal order suppliers and pharmacies, with the latter acting as a subcontractor or intermediary. Most continence care in the Netherlands is currently based on a system where the amount of urine loss determines the patient’s incontinence profile, which dictates the level of reimbursement. Three large Dutch insurance companies (Achmea, VGZ, and CZ) have agreed to use the reimbursement system based on incontinence profile in 2019.^3^

Based on complaints received by the House of Represent-atives, detailing that some patients were not receiving the correct medical aids, in 2015 the Dutch Ministry of Health, Wellbeing, and Sports requested further exploration to improve the status quo.^4^ Taskforces consisting of various stakeholders were therefore established to formulate quality standards for stoma, diabetes, and continence care. Among these, the continence care taskforce developed a new framework based on considering an individual’s activities of daily living when providing continence products. This new framework is in line with an internationally applicable service specification for continence care that was developed by Wagg et al in 2014. They advised that health care providers use a comprehensive assessment of user, product, and usage-related factors to establish the requirements for incontinence material.^5^ This broadly corresponds to the standard for evaluating containment products (ISO 15621:2017).^6^ This comprehensive assessment provides an important foundation to the new framework.^7^

Clients, primary and secondary healthcare providers, insurance companies, suppliers, and manufacturers of continence care products together designed this new framework. In December 2018, the Dutch National Health Care Institute that has responsibility for maintaining the quality, accessibility, and affordability of healthcare added the framework to their register. It contains agreements on the quality of continence care products that are reimbursed in the basic care package.^7,8^ It is compulsory to be insured with a basic care package in the Netherlands, the content of which is determined by the government and can change every year.^9^ However, the impact of the new framework on health care in daily practice is unknown, especially concerning Dutch pharmacy processes. Therefore, we explored the initial expectations of pharmacy staff regarding this new framework and clarified what they considered to be potential facilitators and barriers in continence care.

## Methods

### Research design

We conducted a qualitative study of focus group discussions with pharmacy employees involved in the delivery of continence products (April 2019). This design was chosen to stimulate interaction and group discussion, creating an opportunity for open input on the facilitators, barriers, and expectations related to the new framework based on the participants’ experiences.^10^

### New framework

The new framework describes the process of care regarding continence products for home-dwelling people with continence disorders, as referred to in the Health Insurance Act. The framework is based on providing continence products with an individual’s activities of daily living in mind, seeking to ensure that the provision of continence care products is client-oriented, effective, efficient, and transparent. The new framework has 7 steps: (1) identify the problem; (2) formulate care demand; (3) draft care plan; (4) select, try, and decide; (5) deliver and instruct; (6) use; and (7) evaluate. The new framework also indicates which professionals are involved in which step, divided into primary and secondary care.^11^ Although the framework covers both absorbing and draining products, this study focused on absorbing products.

### Participants

The focus groups were performed next to an observational study on continence care. For this we selected pharmacies that were not familiar with the framework, but that were willing to implement it in their workflow and recruited them by e-mail and/or telephone. Pharmacies that were not willing to participate, did mostly not because of limited continence care, recent changes in staffing or lack of time. We invited each pharmacy that was willing to participated in the observational study to choose and send in 1 or more employees who were actively involved in continence care for a focus group and training to become familiar with the new framework. They could choose between 2 dates and the focus group meetings were held face-to-face prior to this training. The first of the 2 focus groups included eleven participants from 5 pharmacies (3 pharmacists, 7 pharmacist’s assistants, and 1 continence nurse). The second focus group included 4 pharmacist’s assistants from 3 pharmacies. Results of the observational study will be reported elsewhere. Participating pharmacies also differed in size and by region and urbanization.

### Procedure and data collection

The Consolidated Criteria for Reporting Qualitative Studies were applied in the reporting of this study.^12^ The first author (MCS) moderated the focus groups, after receiving training and instructions from an experienced moderator and qualitative researcher (EIM). Although she had mail, phone, or face-to-face contact with the 3 pharmacists about their willingness to participate and about the observational study, she had no prior contact with the other focus group participants.

A semi-structured interview guide was used that comprised the following main topics: (1) delivery of continence products, (2) law and regulations, (3) continence product problems, and (4) the new framework. The guide was developed by the research group, which consisted of researchers and a general practitioner (GP) who was a qualified epidemiologist, all of whom had experience in this field. The topics in the interview guide were structured in a logical and constructive way and were based on the findings of a literature review, information about the new framework, and knowledge of continence care. To establish rapport, interviews started with the non-threatening, gentle question what they expected from the focus group, before moving on to more challenging topics.^13^ In addition to using open questions, we included 2 other assignments to promote discussion: an sticky note assignment in which the participants were asked to individually write down pros and cons of the current continence care and a flip-board assignment in which they were asked to write down the top 3 of problems they run into in daily practice in subgroups.^3^ The same interview guide was used for both focus groups.

### Data analysis

The focus groups were audio and video recorded and transcribed verbatim, after which the transcript was pseudo-anonymized (names and personal characteristics were removed, but not the roles and perspectives that are important for answering the research question). A thematic content analysis was used to analyze the data using ATLAS.ti version 8.4.^14^ The round of open coding was done by 2 authors (MCS and HW) independent of each other, after which the codes were discussed and re-coded until consensus was reached. A codebook was then formulated. Finally, the themes and subthemes identified during the coding process were discussed with all authors until consensus was reached for all themes.

### Ethical considerations

The Medical Ethics Review Committee of University Medical Centre Groningen confirmed that the Medical Research Involving Human Subjects Act (WMO), which includes the Declaration of Helsinki, did not apply to our study. Participants gave informed consent before the start of the focus group.

## Results

### Participants

The focus groups included 15 participants (female: 87%) with a mean age of 36 years (range: 20–50 years) from 7 pharmacies. Excluding planned 20-minute breaks, the first and second focus groups took 110 and 70 minutes, respectively. The participant characteristics are shown in Table 1.

**Table 1.** Participant details by focus group.

Pharmacy	Participant	Sex	Age (years)	Code
Focus group 1				
Pharmacy A	Pharmacist	Male	42	PH-A
	Continence nurse	Female	48	CN-A
	Pharmacist’s assistant	Female	32	PA-A
Pharmacy B	Pharmacist	Male	48	PH-B
	Pharmacist’s assistant 1	Female	30	PA-B1
	Pharmacist’s assistant 2	Female	27	PA-B2
Pharmacy C	Pharmacist’s assistant 1	Female	32	PA-C1
Pharmacy D	Pharmacist’s assistant 1	Female	20	PA-D1
	Pharmacist’s assistant 2	Female	40	PA-D2
Pharmacy E	Pharmacist	Female	43	PH-E
	Pharmacist’s assistant	Female	30	PA-E
Focus group 2				
Pharmacy C	Pharmacist’s assistant 2	Female	35	PA-C2
Pharmacy F	Pharmacist’s assistant 1	Female	31	PA-F1
	Pharmacist’s assistant 2	Female	28	PA-F2
Pharmacy G	Pharmacist’s assistant 1	Female	50	PA-G

### Themes

The main themes derived from the focus groups discussions were (1) “providing continence products,” (2) “practice versus policy,” (3) “actors in continence care,” and (4) “communication.”.

#### Providing continence products

The providing continence products theme was composed of the subthemes “reimbursement system,” “determining continence products,” and “new framework.”

##### Reimbursement system

Working with an incontinence profile, based on the amount of urine loss and dictating the level of reimbursement, gave direction when advising about continence products, but also had some drawbacks. This occurred when participants had to explain that they can only supply a limited number of products a day, when patients wanted more products for hygiene reasons, and when patients needed extra products, such as during a common cold. They also felt that saving on continence products could ultimately cost more money for example when patients get cheaper materials for which they need home care to help them change.

*"I just find it very frustrating that people just cannot change themselves independently, because they are not allowed to wear disposable pants and therefore need home care."* (PA-E)

##### Determining continence products

The participants considered it a challenge to determine the degree of incontinence for the profile because it can be hard to estimate urine loss and because the degree of incontinence can fluctuate.

*"When you ask people how incontinent they are, a droplet or a splash? . . . Some people are very modest and say: 'yes, it is just a splash.' But what's a splash? And if sometimes it’s a splash and sometimes a droplet or a splash, you know, you can assume that what you need in the worst case scenario."* (CN-A)

The participants appreciated the varied choice of continence products, but they found it challenging to compromise between the patients’ needs and desires and the reimbursement value.

##### New framework

Participants were positive about providing continence products based on individual daily activities, as defined in the new framework, and valued the opportunity to provide different products for days with less or more daily activities. An example of this that was mentioned is a patient that can manage incontinence with reimbursed disposable pads when at home, but needs disposable pants when traveling by car for a family visit every other week. Another example is a patient that exercises once a week in which case the reimbursed materials that are normally used are not sufficient.

Participants were positive about the new framework despite the feeling that they already provided continence products tailored to individuals’ needs. However, the participants were not convinced of the applicability of the new framework in daily practice because they expected insurers to persist with the current reimbursement system irrespective of the new framework: *"Yes, but the reimbursement is not yet based on individual daily activities."* (PH-A)

#### Practice versus policy

Participants discussed that daily practice and continence care policies differ, offering several examples. Some patients visit the pharmacy directly without a GP prescription, causing extra work for pharmacies. Other patients may change their behavior (eg, drinking less fluid than advised) because of the limited number of products they can use per day. Others still may use continence products incorrectly, sometimes even based on the advice of home care services.

*"[In the case that people use both disposable pads and pants, where] home care just says: 'put the pad in the pants.' Yes, no it doesn’t work that way . . .. [Then] the health insurance is reimbursing a pair of underpants instead of pants."* (PA-F1)

Participants felt that a patient’s characteristics influenced the behaviors of, and possibilities for, that patient. For example, mixed incontinence or diarrhea necessitated more products or different products, while limited hand function could prevent patients from changing certain products without help or home care. Patients who are older, deaf, or suffer from dementia may have difficulty understanding the system for continence products, as may migrants or patients with language barriers. Obesity or young age may necessitate non-standard product sizes that are more expensive. It was also noted that, due to shame, patients can be incontinent for some time before seeking help. By contrast, younger patients who are more experienced with digital services will be less likely to have problems with the system.

#### Actors in continence care

Participants discussed the facilitators and barriers related to the various stakeholders (actors) involved in the provision of continence care.

##### Insurance providers

Participants felt that insurers caused barriers by having complex policies for continence care and reimbursement (eg, product ordering is only allowed through postal order suppliers, but patients are unaware and go to the pharmacy first): *"Sometimes you don’t pay close attention. Then you're already busy with a patient, before realizing we can’t provide products for this insurer."* (PA-G)

##### Postal order suppliers

With regards to the collaboration between pharmacies and postal order suppliers, processes and communication were highlighted as important themes.

*"Yes, so I have to send another message to the postal order supplier that they have to call that patient, because he/she has problems. And sometimes that takes days. Then that patient comes back to us: 'I haven’t got anything yet and I haven’t been called.' This results in a lot of administrative hassle and ultimately . . . it takes so much longer before [the patient] is helped."* (PH-E )

##### General practitioners

A lack of knowledge regarding continence products and other related treatments was noted for GPs who prescribed the reimbursable products: *"We almost always receive a prescription with a random [product] on it and then we arrange it ourselves with the patient"* (PA-G). This caused frustration with some participants, but others accepted it: *"I understand that the GP doesn’t always have time for that. . . . That they’ll think 'Oh, let the pharmacy do it.' . . . We just know a little bit more about it. So, I think it is positive that we [determine the products]"* (PA-F2).

##### Home care

Home care professionals in the Netherlands are trained nurses or nurse assistants who provide medical and non-medical care, such as assistance with the activities of daily living, for community-dwelling people. Their advice was cited as a cause of confusion because they sometimes recommended products that could not be reimbursed. *" They [homecare] receive continence products from patients that stopped using them or who passed away. They [homecare] share these with other patients but we [the pharmacy] will never supply those products [because they cannot be reimbursed for these patients]"* (PH-A).

##### Government

The general rules and reimbursement policies set by the governmental were also mentioned, such as the eligibility for reimbursement (eg, only chronic incontinence is reimbursed). *"Urinary incontinence has to last for at least two months and fecal incontinence for two weeks,. . . "* (CN-A).

#### Communication

Knowledge of how continence care is organized and how the reimbursement system works were important to communication between pharmacy staff and patients. It was emphasized that the system is hard for patients to understand: *"It’s a difficult subject to explain; all those steps are quite difficult"* (PH-A). Participants further commented that the reimbursement conditions set by insurers were not clear to patients, with there often being differences in the information given to patients and pharmacies. When patients had questions about reimbursement, they frequently visited a pharmacy in preference to asking their insurer. Similarly, pharmacy staff often facilitated communication between patients and postal order suppliers, which was cited as causing extra work for pharmacies. However, pharmacies that invested in their relationship with local home care professionals reported that they experienced improved cooperation.

## Discussion

### Main findings

In this focus group study, we aimed to explore the initial expectations of pharmacy staff about a new framework for continence care and its implementation. To gain a better understanding of how to improve these, we also sought to establish the perceived facilitators and barriers related to continence care in the current situation. The participants supported the fundamental idea of the new framework to take individual daily activities into account when providing continence products. However, they were not convinced of its applicability in daily practice, especially because they expected insurance companies to retain the current reimbursement system despite the new framework. Information from beyond this qualitative study has confirmed this expectation, with no changes evident in health insurer practices even though the new framework should now be in use.^15^

One of the main barriers to continence care was the low reimbursement for the incontinence profiles, which limited the freedom of participants to provide continence products tailored to the needs of patients. This also resulted in barriers when practice did not comply with policies. Wider margins for product reimbursement will enable pharmacy employees to take individual daily activities into account when providing products, especially when there are variations in the amount of material needed (eg, when there are varying levels of incontinence or activity).

The lack of communication between the various stakeholders in continence care was another important barrier that caused frustration and extra work for pharmacy staff. Clear communication could save time and reduce these frustrations, and it will prove essential for implementing the new framework. Uniformity and clarity in the information provided by health insurers and postal order suppliers to patients and professionals is an essential starting point. It was also notable that participants felt that GPs should limit themselves to prescribing by indication, leaving product choice to the pharmacies. Concerning home care professionals, it may be helpful to invest in knowledge transfer regarding the possibilities within the new framework and the corresponding reimbursement given the positive experiences in pharmacies that had already invested in relationships with local professionals. Given that the role of home care professionals was raised multiple times, it was evident that pharmacy employees considered these important actors.

### Comparison with the literature

We found that our participants supported the idea of tailoring patient needs based on a comprehensive assessment of user, product, and usage-related factors, consistent with their current practice of tailoring care where possible. Nevertheless, our participants expressed fear that insurers will not change their reimbursement system despite the new framework being included by the Dutch Register of the National Health Care Institute since December 2018. This is consistent with our experiences in routine practice.

Wagg et al^5^ discovered a decreasing trend in healthcare spending on continence care in their research, consistent with our findings that participants experienced challenges in delivering continence products within the reimbursement limits. Urinary incontinence is known to incur a substantial economic burden.^16^ The direct costs of urinary incontinence include those of treatment, managing the condition at home, and buying continence products.^17^ Our participants mentioned that saving on incontinence materials can increase costs in other health care domains, such as home care, thereby potentially making any cost savings a false economy. Cost utility analyses should be done to investigate this further.

Research has emphasized the importance of communication about continence treatment between healthcare providers. Such an approach should help to avoid gaps in care and is key to optimizing the management and treatment of urinary incontinence.^4,18^ This was reported by our participants, who added that communication with insurers, postal order suppliers, and GPs were all important. Unfortunately, several studies show that GPs do not always follow guidelines or that they lack awareness and knowledge of continence care,^14,19^ often resulting in substandard care. This is consistent with our participants’ experiences that GPs lack knowledge about continence products and related treatments, which was perceived to be a barrier.

Participants had mixed feelings regarding home care professionals because collaboration was not optimal and advice offered by them was often incorrect. The same was observed in a Swedish study,^20^ where nurses who provided continence products had mixed feelings concerning the healthcare assistants who either had great insights into the patient’s situation or who gave incorrect advice (eg, overly large products or using multiple products at the same time).^20^ According to Hunter and Wagg,^21^ continence care is not always a priority for nurses, with many often lacking specific knowledge about this topic. This may explain the incorrect advice given by home care professionals in the Netherlands.

### Strengths and limitations

We included participants from pharmacies that differed in size and by both region and urbanization. The participants also held a range of different positions within the pharmacy (eg, pharmacists, pharmacist’s assistants, and a continence nurse). Eighty-seven percent of the participants was female, what is comparable with the proportion of 90% in the population of pharmacy employees in the Netherlands.^22^ This sample is a strength because, despite the small sample size, it facilitates the transferability and broader applicability of our results. Another strength was the openness of the focus group discussions, as evidenced by participants raising the topic “home care,” even though it was not a predetermined topic in the interview guide.

A limitation of the first focus group, which included participants of different positions, is that the hierarchical relationships may have caused participants to feel less free to discuss their opinion. However, the impact of this limitation is probably small because the second focus group did not have that limitation and the 2 focus groups had comparable outcomes. Selection bias may also have occurred during the inclusion of each pharmacy, because those with an interest in continence care may have been more inclined to participate in the project than those with no interest. Therefore, our participants may have been more open to new developments, and in particular, the new framework.

## Conclusion

To the best of our knowledge, this is the first qualitative study to have explored continence care provision by Dutch pharmacies. We found that pharmacy employees are positive about the idea of the new framework to provide continence products based on individual daily activities, but that some reservations persist about the feasibility of implementing the framework in daily practice. Notable among the current barriers to continence care are the limited options for reimbursement and the lack of communication. Participants expect that the new framework will fail to generate the required change.

## Implications for Practice

Continence care and its processes and policies are complex. Effective policy making relies on accurate insights from stakeholders into the expectations for the new framework and the perceived facilitators and barriers to implementation. Besides patients, providers of continence products are important stakeholders for their role in the execution of the framework. Given the reservations of the pharmacy employees about the feasibility of implementing the framework in daily practice, the current reimbursement system should be critically reviewed to enable the provision of continence products by individual need and level of activity. Besides that, more effort is needed to change the policies and information provided by all stakeholders in continence care, particularly ensuring that these are more uniform, before the new framework will make a real impact in clinical practice.
